# In vitro study of combined cilengitide and radiation treatment in breast cancer cell lines

**DOI:** 10.1186/1748-717X-8-246

**Published:** 2013-10-23

**Authors:** Tim Lautenschlaeger, James Perry, David Peereboom, Bin Li, Ahmed Ibrahim, Alexander Huebner, Wei Meng, Julia White, Arnab Chakravarti

**Affiliations:** 1 Department of Radiation Oncology, The Ohio State University, Columbus, OH 43210, USA; 2 Comprehensive Cancer Center, The Ohio State University, Columbus, OH 43210, USA; 3 Burkhardt Brain Tumor and Neuro-Oncology Center, Cleveland Clinic, Cleveland, OH 44195, USA; 4 Pharmaceutical Industries Division, National Research Center, Cairo, Dokki 12622, Egypt

**Keywords:** Cilengitide, Breast cancer, Brain metastasis, Radiation

## Abstract

**Background:**

Brain metastasis from breast cancer poses a major clinical challenge. Integrins play a role in regulating adhesion, growth, motility, and survival, and have been shown to be critical for metastatic growth in the brain in preclinical models. Cilengitide, an αvβ3/αvβ5 integrin inhibitor, has previously been studied as an anti-cancer drug in various tumor types. Previous studies have shown additive effects of cilengitide and radiation in lung cancer and glioblastoma cell lines. The ability of cilengitide to enhance the effects of radiation was examined preclinically in the setting of breast cancer to assess its possible efficacy in the setting of brain metastasis from breast cancer.

**Methods:**

Our panel of breast cells was composed of four cell lines: T-47D (ER/PR+, Her2-, luminal A), MCF-7 (ER/PR+, Her2-, luminal A), MDA-MB-231 (TNBC, basal B), MDA-MB-468 (TNBC, basal A). The presence of cilengitide targets, β3 and β5 integrin, was first determined. Cell detachment was determined by cell counting, cell proliferation was determined by MTS proliferation assay, and apoptosis was measured by Annexin V staining and flow cytometry. The efficacy of cilengitide treatment alone was analyzed, followed by assessment of combined cilengitide and radiation treatment. Integrin β3 knockdown was performed, followed by cilengitide and radiation treatment to test for incomplete target inhibition by cilengitide, in high β3 expressing cells.

**Results:**

We observed that all cell lines examined expressed both β3 and β5 integrin and that cilengitide was able to induce cell detachment and reduced proliferation in our panel. Annexin V assays revealed that a portion of these effects was due to cilengitide-induced apoptosis. Combined treatment with cilengitide and radiation served to further reduce proliferation compared to either treatment alone. Following β3 integrin knockdown, radiosensitization in combination with cilengitide was observed in a previously non-responsive cell line (MDA-MB-231). Clonogenic assays suggested little radiosensitization effects of cilengitide.

**Conclusions:**

Cilengitide appears to enhance radiation response in preclinical models of breast cancer. These data suggest that the combination of radiation therapy and cilengitide may prove to be effective where radiation is utilized for the treatment of gross disease in breast cancer, such as in the setting of brain metastasis.

## Background

Brain metastasis from breast cancer occurs in approximately 5% of patients overall, and in 10-16% of patients with metastatic disease [1]. Incidence is thought to be on the rise, as systemic therapy advances lead to better local tumor control and improved survival. Current treatments for these metastases include whole brain radiotherapy, surgery, stereotactic radiosurgery, and chemotherapy [2]. Outcomes for these patients are poor with median survivals ranging from 3.4-25.3 months based on the Graded Prognostic Assessment (GPA) [3]. Radiation is commonly used in the treatment of brain metastasis from breast cancer, where there can be gross disease present in the brain. Given that a significant percentage of these patients succumb to their metastatic disease and demonstrate local progression of their disease in the brain, we sought to investigate agents that may demonstrate additive or synergistic effects with radiation in the setting of breast cancer.

Integrins play a role in regulating cell-extracellular matrix interactions as well as cell signaling pathways that regulate adhesion, growth, motility, and survival [4]. Integrins are expressed on endothelial cells, and play an important role in angiogenesis [5], but have also been identified on a number of cancer cell types [6-8]. αVβ3 integrins specifically have been identified to play a direct role in tumor cell growth as well as invasion and metastasis [9,10]. αVβ3 integrins were shown to be critical for metastatic growth of breast cancer cells in the brain [11]. Therefore, we further examined targeting integrin signaling in combination with radiation.

Cilengitide is a cyclic RGD containing pentapeptide that targets αVβ3 and αVβ5 integrins [12]. This inhibitor has been shown to block glioma cell growth via cell detachment and induction of apoptosis in an in vitro model [13]. In vivo, cilengitide has been shown to inhibit metastatic bone colonization by the breast cancer cell line MDA-MB-231 [14].

In the context of radiation therapy, combination treatment with cilengitide has been shown to radiosensitize lung cancer cell lines [15], with lung cancer representing the tumor site with the highest incidence of metastasis to the brain [1]. Combination therapy of cilengitide and radioimmunotherapy with an L6 antigen targeting antibody conjugated with the beta-emitter ^90^Y has shown to improve outcomes of primary breast tumors in a xenograft model of breast cancer [16]. The combination of cilengitide and external beam radiotherapy has yet to be studied in the context of breast cancer.

We therefore set out to determine if a combination therapy of cilengitide and radiation could be of benefit for breast cancer brain metastases patients. To this end we tested the effect of cilengitide in combination with radiation in a panel of breast cancer cell lines, among them cell lines that have previously been used to study brain metastases from breast cancer.

## Methods

### Cell culture and drug treatment

T-47D (ER/PR+, Her2-, luminal A), MCF-7 (ER/PR+, Her2-, luminal A), MDA-MB-231 (TNBC, basal B), MDA-MB-468 (TNBC, basal A) cell lines cells were purchased from ATCC. T-47D (RPMI-1640 medium supplemented with 10% fetal bovine serum, 1% penicillin/streptomycin, and 0.2Units/ml bovine insulin) and MCF-7 (Eagle’s minimum essential medium supplemented with 10% fetal bovine serum, 1% penicillin/streptomycin, and 0.01 mg/ml bovine insulin) cell lines were maintained at 37°C in a 5% CO_2_ atmosphere. MDA-MB-231 and MDA-MB-468 (Leibovitz’s L-15 medium supplemented with 10% fetal bovine serum and 1% penicillin/streptomycin) cell lines were maintained at 37°C in an air atmosphere. Cilengitide was acquired through the Cancer Therapy Evaluation Program (CTEP) from Merck. Irradiation was performed using a RS-2000 Biological Irradiator (Rad-Source) with 160 kV x-rays with a 0.3 mm copper filter in place at a dose rate of approx. 1.2 Gy/min. All statistical comparisons were performed using repeated measures ANOVA with a Tukey’s multiple comparison test. All research (non-human subject research, non-animal research) was done in accordance with institutional guidelines.

### Western blotting

Untreated cells were washed and trypsinized, followed by pelleting and freezing at -80°C. Upon thawing cells pellets were resuspended in RIPA lysis buffer (50mM Tris–HCl, pH 8.0, with 150mM sodium chloride, 1.0% Igepal CA-630 (NP-40), 0.5% sodium deoxycholate, and 0.1% sodium dodecyl sulfate) supplemented with the addition of Protease inhibitor cocktail (Sigma), phosphatase inhibitor cocktail 1 (Sigma), phosphatase inhibitor cocktail 2 (Sigma), and phenylmethylsulfonyl fluoride (Sigma). Protein concentration was determined with a BCA Protein Assay kit (Thermo Scientific) and 50ug was loaded per well in a 4-20% TGE precast gel (Bio-rad), followed by transfer to PVDF membranes. Membranes were blocked using 5% bovine serum albumin in TBS-T, followed by overnight incubation at 4°C with primary antibody solution for β3 or β5 Integrin (1:1000, cell signaling). Blots were incubated with anti-rabbit secondary antibody (1:2000, cell signaling) was at RT for 1 hr. Blots were then developed using Immobilon Western Chemiluminescent HRP Substrate (Millipore) in the VersaDoc imaging system (Bio-rad).

### Flow cytometry - apoptosis

Apoptosis assays were performed using the Alexa Fluor^®^ 488 Annexin V/Dead Cell Apoptosis Kit (Life Technologies) according to the manufacturer’s instructions. Briefly, cells were plated and allowed to attach overnight. The following morning, cells were treated with indicated cilengitide doses and allowed to incubate for 48 hours. Apoptotic induction controls were treated with 16.6 uM cisplatin 24 hours before staining. After 48 hours, cells were harvested (floating and attached cells) and stained according to manufacturer’s instructions. Single stained and unstained controls were included. Flow cytometry was performed on a BD Facs LSR II flow cytometer under guidance from flow cytometry core staff. Samples were analyzed using Flow Jo software.

### Clonogenic assay

All cell lines were trypsinized and counted, followed by dilution to appropriate levels. Cells were seeded in triplicate in 2 ml growth media into 6-well tissue culture dishes and allowed to attach overnight. The following morning, the indicated doses of cilengitide were added to the cells. Irradiation was performed at the indicated doses 1 hour after drug treatment. Cells were then maintained at 37°C in the appropriate atmosphere for 10 days to 1 month until appropriate sized colonies had formed. Once colonies were visible, cells were stained in 0.5% crystal violet in methanol for 2 hours. Colonies were counted with the use of a dissecting microscope with a cutoff of 50 cells. Dose enhancement ratios are reported for 37% cell survival.

### Cell counting

Cells were plated at 2 × 10^5^ cells/well and allowed to attach. Cell counting was performed following 1 hr treatment with the indicated cilengitide doses. Detached cells were removed and wells were washed with PBS. Remaining attached cells were then trypsinized and diluted 1:2 with trypan blue. Live cells were counted in duplicate using the Nexelcom Cellometer Auto T4 cell counter (Nexelcom Bioscience). Remaining cell viability was at least 95%.

### MTS assay

Proliferation assays were performed using the CellTiter Aqueous One Solution Cell Proliferation Assay (Promega) according to modified manufacturer’s instructions. 2 × 10^4^ cells/well were plated in 500 ml growth medium in triplicate. Cells were treated at the indicated dose of cilengitide on day 0. Control readings were measured on day 0 to control for plating inaccuracies and cell metabolism differences. The CellTiter solution was diluted 1:6 and 240 ul/ well was added. Plates were incubated for 3 hrs at 37°C, followed by removal of 100 ul of MTS solution. Absorbance at 490 nm was obtained using a Mithras LB 940 plate reader (Berthold Technologies). Treated cells were left for 96 hours and absorbencies were obtained as above. ITGB3 knockdowns were performed using ON-TARGET Plus SMARTpool Human ITGB3 siRNA and Control Non-Targeting siRNA. Cells were treated with 25 nM siRNA for 48 hours before plating. Day 4 values were controlled to Day 0 values within each cell line. Values were also controlled to untreated cell values and reported as % Cell Viability compared to untreated cells. For the ITGB3 knockdown experiments, normalization was performed against the untreated control cells for each group (Non-Targeting cells or ITGB3 knockdown cells respectively).

## Results

### Cilengitide target expression in breast cell lines

**Figure 1:**
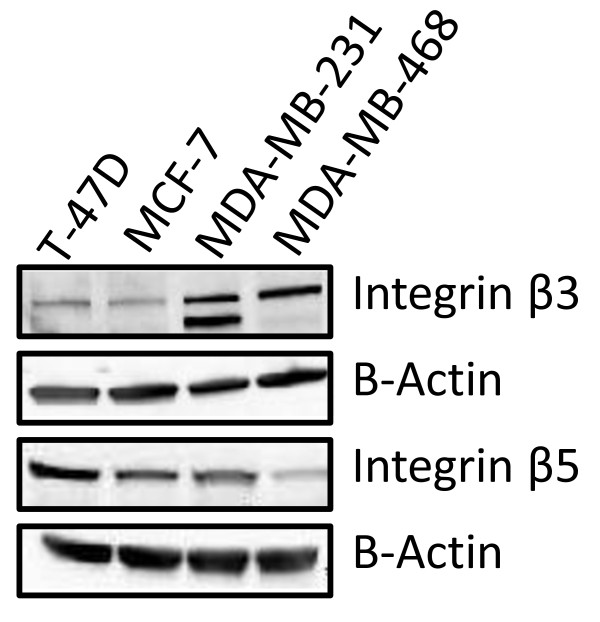
**Expression of β3 and β5 integrin in a panel of breast cancer cell lines*. ***Western Blot of T-47D, MCF-7, MDA-MB-231, and MDA-MB-468 cell lines for β3 and β5 Integrin. β3 target is observed in all cell lines, with MDA-MB-231 and MDA-MB-468 showing the highest levels, while T-47D and MCF-7 cells showed a lower level of expression. β5 target is observed in all cell lines, with T-47D showing the highest levels, MCF-7 and MDA-MB-231 showing intermediate levels, and MDA-MB-468 showing a lower level of expression.

β3 and β5 integrins, found as dimers with αV integrins, are the targets for the inhibitor cilengitide. αVβ3 integrin is thought to represent the primary target, while αVβ5 is an alternate target. We examined the breast cancer cell lines T-47D, MCF-7, MDA-MB-231, and MDA-MB-468 for β3 and β5 integrin expression by western blot. As shown in Figure 1, we detected both β3 and β5 integrin in all of our cell lines (β3 integrin can be detected as possibly 3 different bands of 97, 110, and 130 kDa). T-47D and MCF-7 cell lines expressed β3 integrin at low levels, while MDA-MB-231 and MDA-MB-468 cell lines expressed β3 integrin at higher levels than the other two cell lines studied. β5 integrin was most strongly expressed in T-47D cells, at intermediate levels in MCF-7 and MDA-MB-231 cells, and only very lowly expressed in MDA-MB-468 cells.

### Effects of cilengitide alone

#### ***Cell detachment***

**Figure 2:**
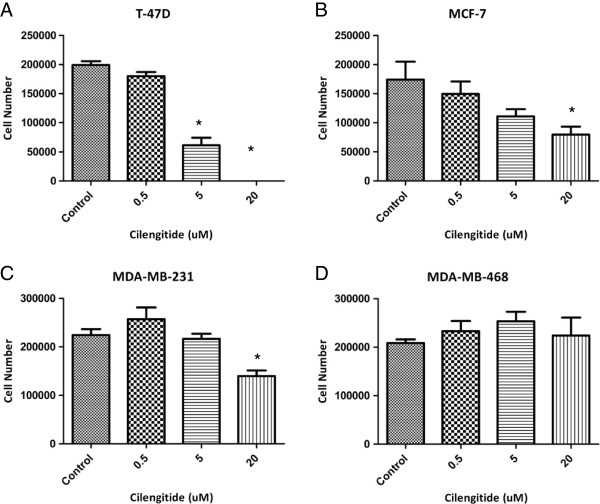
**Cell detachment following 1 hr Cilengitide treatment*. ***Cells were treated with cilengitide for 1 hr, washed with PBS, and remaining attached cells were trypsinized and counted. **A)** T-47D cells showed a strong dose response, with almost complete cell detachment at 20 uM cilengitide treatment. **B)** MCF-7 cells showed moderate cell detachment similar to **C)** MDA-MB-231 cells. **D)** MDA-MB-468 cells showed little to no cell detachment following this short exposure to cilengitide. Figures show Mean ± SEM and represent the average of three experiments. * = ≤ 0.05.

Once expression of the target of cilengitide was verified, we assessed the effects of cilengitide treatment as a single therapy in our panel of cell lines. Previous studies have shown that cilengitide causes cell detachment in culture [13,15]. The role of integrins in cell adhesion to uncoated plastic dishes is probably related to serum vitronectin adhering to the plastic [17]. We wanted to examine this cellular detachment in our panel of cell lines. Briefly cells were treated with cilengitide (500 nM, 5 uM, and 20 uM) for 1 hour. Detached cells were then washed away with PBS, and remaining attached cells were trypsinized and counted. We observed that in most of our cell lines there was a dose dependent effect of cilengitide on cell detachment. At the highest dose tested (20 uM) T-47D cells showed the most marked response, with almost complete detachment of cells following 1 hour of treatment (Figure 2). MDA-MB-468 cells showed little to no cell detachment at this early timepoint, even at this highest dose. Both of the other cell lines tested showed a moderate response to the 20 uM dose, losing about 50% of plated cells (Figure 2). Most of these cells, however, are likely to be merely detached, as opposed to dead. Cells treated for 1 hour with 20 uM cilengitide to cause detachment, were then washed with PBS and re-plated into a new cell culture dish. At least a part of these washed cells were able to attach and grow normally (data not shown). Cells left in cilengitide up to 96 hours remained detached (data not shown).

#### ***Growth inhibition***

**Figure 3:**
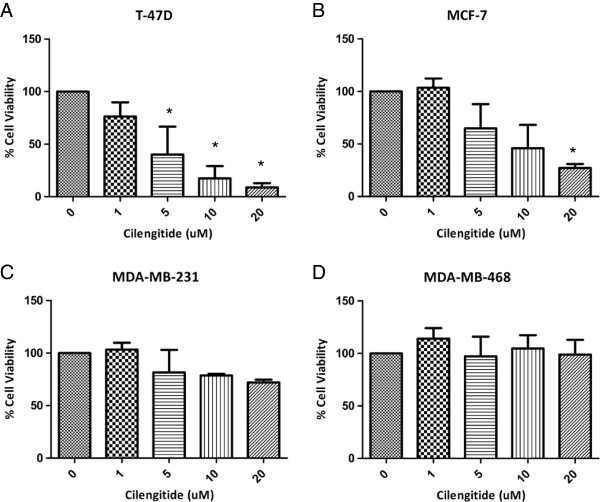
**MTS proliferation assay following prolonged cilengitide treatment*. ***Cells were treated with Cilengitide for 96 hours, followed by washing and addition of an MTS substrate. After 4 hour incubation, absorbance was read and cell viability as percent of control treated cells was reported. **A)** T-47D cells show the most marked response, with little to no cell growth at the 20 uM cilengitide dose. **B)** MCF-7 cells show a moderate growth delay. **C)** MDA-MB-231 cells also show a moderate growth delay. **D)** MDA-MB-468 cells show little to no growth delay compared to controls at this 96 hour timepoint. Most cell lines showed a dose response to cilengitide **(A**-**C)** with only MDA-MB-468 cells showing no effects. Figures show Mean ± SEM and represent the average of three experiments. * = ≤ 0.05.

The same trends discussed above were observed in our panel of cell lines in a longer term proliferation assay. Breast cancer cell lines were plated, allowed to attach overnight, and then treated the following morning with varying doses of cilengitide (1 uM, 5 uM, 10 uM, and 20 uM). The cells were then allowed to grow for 96 hours in the presence of cilengitide and cell viability was determined. As shown in Figure 3 the cell responses at 96 hours following cilengitide treatment are similar to the early 1 hour detachment response. In this assay, T-47D cells still showed the most marked response, with almost complete cell loss compared to untreated cells at the 20 uM dose (Figure 3). MDA-MB-468 cells again showed no response to cilengitide treatment, even at the 20 uM dose. In this assay, MDA-MB-231 cells had a reduced response when compared to the previous early cell detachment assay, showing only 25% reduction in cell proliferation after 96 hours of cilengitide exposure compared to controls as opposed to 50% of cells detached after 1 hour (Figures 2C and 3C). On the other hand MCF7 cells showed a more noticeable response, with nearly an 80% reduction in cell proliferation compared to the 50% of cells lost after 1 hr of treatment (Figures 2B and 3B). Overall these results are consistent with the early cell detachment results. Cilengitide appears to have a significant effect, on both cell attachment and cell growth, in our breast cancer cell lines.

#### ***Cilengitide induces apoptosis***

**Figure 4:**
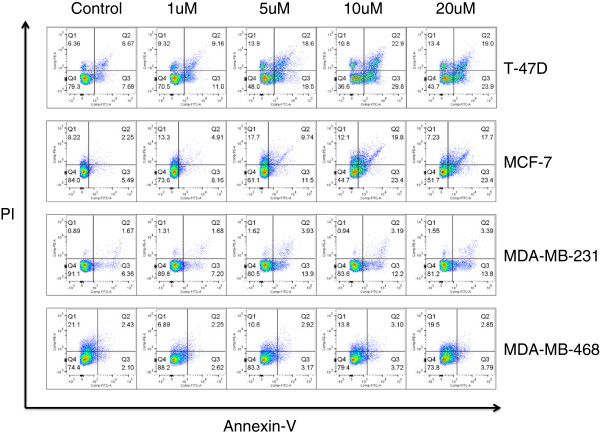
**Cilengitide induces apoptosis in breast cell lines*. ***Cells were treated for 48 hours with the reported cilengitide doses and then harvested for Annexin V/PI staining and analysis by flow cytometry. T-47D cells showed the highest level of apoptotic/dead cells. MCF-7 cells showed the next highest levels of apoptotic/dead cells, with levels very similar to T-47D cells. MDA-MB-231 cells show a moderate induction of apoptosis. MDA-MB-468 cells show no apoptotic induction, which mirrors the cell detachment and proliferation assay results. Experiment was performed three times with similar results. Figures are from a representative experiment.

To examine the mechanism by which cilengitide reduces viability in our cells, we performed an apoptosis assay at an intermediate timepoint (48 hours) following cilengitide treatment. First, cilengitide treatment alone induced apoptosis in all cell lines except for the MDA-MB-468 cells, and this apoptotic induction was dose dependent (Figure 4). This pattern of cell line dependent cilengitide responsiveness is consistent with our earlier cell detachment results (Figure 2) and cell proliferation data (Figure 3). In this assay, we again observed that the most significant effects of cilengitide occurred in the T-47D cell line. The late apoptotic/dead (Q2) and early apoptotic (Q3) events were increased from Q2 – 6.67%, Q3 – 7.69% in T-47D control cells to Q2 – 19.0%, Q3 – 23.9% in cells treated with 20 uM of cilengitide. MCF-7 cells showed the next highest response (control; Q2 – 2.25%, Q3 - 5.49% to 20 uM; Q2 – 17.7%, Q3 – 23.4%). MDA-MB-231 (control; Q2 – 1.67%, Q3 – 6.36% to 20 uM; Q2 – 3.39%, Q3 – 13.6%) showed a more moderate effect of cilengitide treatment in this assay. MDA-MB-468 cells showed no response to cilengitide, as in the other assays performed (control; Q2 – 2.43%, Q3 – 2.10% to 20 uM: Q2 – 2.85%, Q3 – 3.79%). Our findings indicate that cilengitide is able to induce apoptosis and death in some of our tested breast cancer cell lines when used as a single treatment. This finding is in line with other reports which indicate that cells detached by cilengitide treatment go on to apoptose [13]. However, even in a cell line/cilengitide dose combination (T-47D and 20 uM) where 100% of cells appear detached microscopically after one hour (data not shown, compare to Figure 1) there are still approximately 44% of live cells (Quadrant 4) at 48 hrs. It was also observed in T-47D cells exposed to cilengitide for 96 hours that following washing cells were able to attach and grow (data not shown) indicating that even at 96 hours, not all of the detached cells are dead or dying.

### Effects of cilengitide in combination with radiation

**Figure 5:**
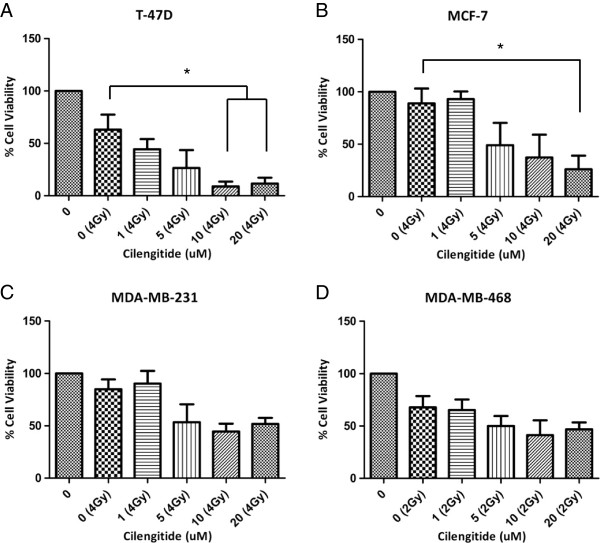
**MTS proliferation assay following prolonged cilengitide treatment in combination with ionizing radiation*. ***Cells were treated in the same manner as in Figure 3, with the addition that for the radiation groups, cells were treated with the indicated dose of IR following 1 hr of cilengitide treatment. All four cell lines show a combination effect where cilengitide in combination with radiation has an enhanced growth delay effect when compared to IR alone treated cells. **A)** T-47D cells show this combination effect at lower doses of cilengitide than other cells lines. **B)** MCF-7 cells show an improvement over radiation alone with cilengitide combination. **C)** MDA-MB-231 cells show a trend toward an enhanced effect of cilengitide in combination with IR treatment. **D)** MDA-MB-468 cells also show only a trend toward increased growth delay in the combination groups versus the radiation alone group. Figures show Mean ± SEM and represent the average of three experiments. * = ≤ 0.05.

**Figure 6:**
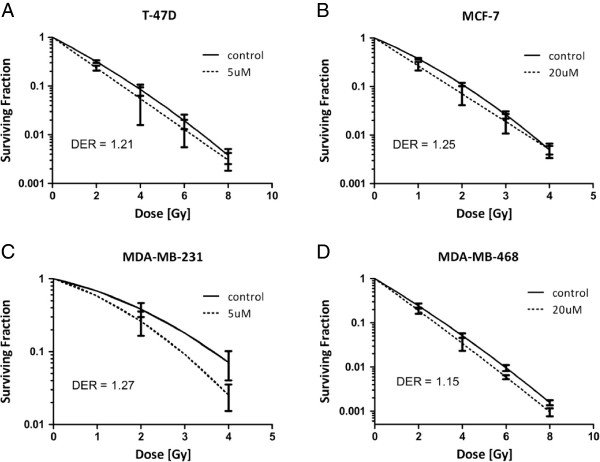
**Clonogenic assay in combination with cilengitide in breast cell lines*. ***Clonogenic assays were performed with our panel of breast cell lines. Cells were plated at specific cell numbers and treated with indicated doses of cilengitide. After 1 hr of treatment, cells were irradiated and then incubated for up to 4 weeks to allow colony formation. Cells were then stained and counted. All four cell lines show small dose enhancement ratios with the addition of cilengitide, indicating no or very little radiosensitization effects. **A)** T-47D (DER – 1.21), **B)** MCF-7 (DER – 1.25), **C)** MDA-MB-231 (DER – 1.27), **D)** MDA-MB-231 (DER – 1.15). Figures represent the average of three experiments.

After characterizing the response of our cell lines to cilengitide as a single therapy, we sought out to determine if cilengitide could serve as a radiosensitizer. First, we performed a proliferation assay (MTS) as discussed earlier (Figure 3) with ionizing radiation (IR) added to the cilengitide treatment groups. T-47D, MCF-7, and MDA-MB-231 cells were treated at 4 Gy, while MDA-MB-468 cells were treated at 2 Gy. An initial test had shown that MDA-MB-468 cells were especially sensitive in terms of viability at 96 hours after IR with 4 Gy (data not shown). All of the cell lines appeared to have reduced viability after combination treatment of cilengitide and IR compared to IR alone (Figure 5). For T-47D and MCF-7 cells those differences were statistically significant, while for MDA-MB-231 and MDA-MB-468 we observed only a trend towards improved efficacy of the combination treatment. Clonogenic assays showed only very minor increases in radiosensitivity following cilengitide treatment in some of the cell lines tested (Figure 6). These results indicate that cilengitide is enhancing the effects of radiation in our panel of cells, and that there may be an additive effect for this combination treatment.

**Figure 7:**
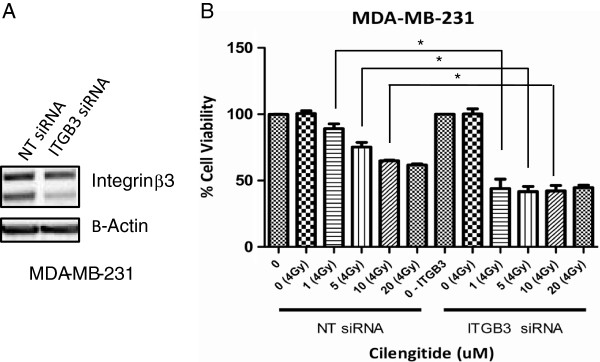
**MTS proliferation assay following ITGB3 knockdown in MDA-MB-231 cells*. ***Cells were treated with 25nM siRNA (either NT or ITGB3) for 48 hours before being treated in the same manner as described in Figure 5. **A)** Target knockdown was confirmed via western blot. **B)** Cells treated with NT siRNA showed a similar pattern to previous results, while cells treated with ITGB3 siRNA showed a pronounced radiosensitization in combination with cilengitide treatment. NT untreated controls and ITGB3 untreated controls were used for normalization within their respective groups. The ratio of ITGB3 knockdown/NT treated cell viability cell viability at 96 hours is 0.935. Figures show Mean ± SEM and represent the average of three experiments. * = ≤ 0.05.

It was observed that the least responsive cell lines in this study were those with the highest levels of β3 and lowest levels of β5 integrin target. The association of high expression levels of integrin β3 and poor response to cilengitide in our small cell line panel suggests that integrin β3 is the relevant cilengitide target. One possibility for these results is that this target is not completely inhibited at the cilengitide doses used. Since integrin β5 expression was associated with responsive cell lines, we determined that this target was likely being adequately inhibited by the cilengitide doses studied. To examine if these cells were unresponsive to cilengitide treatment due to incomplete β3 blockade at tested treatment doses, β3 integrin was knocked down in the MDA-MB-231 cell line. Approximately 50% knockdown was achieved in these cell lines (Figure 7A). 48 hours following knockdown, cells were plated and treated as in the above experiments. Figure 7B shows that cells with reduced β3 integrin show enhanced response to radiation and cilengitide combination treatment, while previously they were minimally responsive to cilengitide and radiation treatment (Figure 5).

## Discussion

Brain metastases from breast cancer occur in 10-16% of patients who eventually develop metastatic disease [1]. The advent of brain metastases often is a life altering event for breast cancer patients and not uncommonly impacts quality of life and cognitive functioning. Outcomes for patients with brain metastases are poor with median survival times ranging from 3.4-25.3 months depending on GPA [3]. There is an urgent need for novel therapeutic approaches to improve outcomes in this patient population.

In this study we present our findings suggesting cilengitide treatment can induce cellular detachment and apoptosis, and reduce proliferation in a panel of breast cancer cell lines. While this is, to our knowledge, the first report to describe cilengitide effects in combination with radiation in a panel of breast cancer cell lines our findings are consistent with previous reports describing induction of apoptosis, reduced proliferation, and cellular detachment after cilengitide treatment in glioma [13] and lung cancer [15] models.

The primary target of cilengitide is αvβ3 integrin. Integrin expression on tumor cells plays a role in activating many of the major cell survival pathways including ERK and PI3K signaling [18]. These pathways are known to lead to increased tumor cell proliferation, migration, and resistance to apoptosis [19]. Furthermore inhibition of those pathways can result in radiosensitizing effects in many cancer cell lines [20]. Therefore, we speculate that these pathways could mediate the treatment effects in breast cancer cell lines as well, and it is not surprising that the integrin inhibitor cilengitide has anti-tumor activity in lung and glioma models or in breast cancer models as here presented.

The magnitude of cilengitide effects on breast cancer cell lines, however, was fairly heterogeneous among the cell lines tested. Given that only four cell lines were tested no definitive conclusions can be drawn at this time as to which breast cancer brain metastases patient subpopulations might derive the most benefit from cilengitide containing treatment regimens. It appeared that cell lines that are ER/PR positive and Her2 negative or cell lines with lower expression levels of integrin β3 had the most significant response to cilengitide treatment in this study. Experimentally reducing the expression of integrin β3 in a cell line with high integrin β3 expression (MDA-MB-231) resulted in increased efficacy of cilengitide and radiation. Given that integrin β3 knockdown can sensitize cells to the tested cilengitide doses, it appears that this target is not completely inhibited in cell lines with high expression of integrin β3. While it is still possible that integrin β5 inhibition is responsible for the enhanced response seen in the T-47D or MCF-7 cell lines, these results indicate that at least a portion of the cilengitide resistance in our other cell lines is due to the high levels of integrin β3 expression observed. While the results of our proliferation assays have shown clear statistical benefit of combination therapy compared to either therapy alone in some breast cell lines, our clonogenic data shows little to no formal radiosensitization effect. Our results indicate a more significant benefit of combined radiation and cilengitide treatment at earlier timepoints compared to the long term results of the clonogenic assays. This difference could well be due to the different timepoints tested. However, the performed normalization of the radiated arm to the cilengitide alone treated cells likely contributes to this observed difference as well. Our data however is similar to reports using glioma models [21] where modest radiosensitizing effects of cilengitide were found. Previously it has been reported that cilengitide and radiation have additive effects in glioma cell lines [22]. Our data indicate a similar additive nature of combination cilengitide and radiation therapy in breast cancer cell lines, rather than a synergistic or supra-additive effect. These results are more limited in scope when compared to more significant radiosensitizing effects of cilengitide reported in NSCLC models [15]. The underlying reasons for the differences in potency of cilengitide as a radiosensitizer in different cancers remain to be identified and is subject to further studies. Despite the differences in efficacy seen comparing our assays, combined cilengitide and radiation treatment appeared to be beneficial for most of the tested breast cancer cell lines.

In summary, our data suggest cilengitide treatment has anti-tumor activity in breast cancer cell line models, and that combination treatment with radiation appears to affect cells more than either treatment alone. Given that some of the cell lines used in our study have previously been used as breast cancer brain metastases models [23], and that cilengitide potentially can achieve therapeutically relevant concentrations in the brain, we conclude that combined cilengitide and radiation treatment could be a promising therapeutic strategy for a subset of breast cancer brain metastases patients.

## Conclusions

Combined cilengitide and radiation therapy appears to be more efficacious than either treatment alone in breast cancer cell lines. This study, along with previous work highlights the promise of cilengitide therapy in combination with radiation. Based on the ability of cilengitide to accumulate in the brain combination treatment of cilengitide and radiation could be beneficial clinically for a subset of breast cancer brain metastases patients.

## Abbreviations

GPA: Graded prognostic assessment; CTEP: Cancer therapy evaluation Program; IR: Ionizing radiation.

## Competing interests

The authors declare that they have no competing interests.

## Authors’ contributions

TL conceived of the study, and participated in its design and coordination and helped to draft the manuscript. JP participated in the design of the study, participated in the Western blot analysis and performed apoptosis, proliferation, and clonogenic survival assays, and helped draft the manuscript. DP conceived of the study, and revised the manuscript critically for intellectual content. BL participated in the cell culture studies, and participated in drafting the manuscript. AI participated in Western blot analysis and participated in revising the manuscript. AH participated in the design of the study, participated in statistical analysis, and participated in drafting the manuscript. WM participated in the design of the study, and participated in drafting the manuscript. JW participated in designing of the study, and revised the manuscript critically for intellectual content. AC conceived of the study, and participated in coordination of the study, and revised the manuscript critically for intellectual content. All authors read and approved the final manuscript.
